# Absence of Landau damping in driven three-component Bose–Einstein condensate in optical lattices

**DOI:** 10.1038/s41598-018-29454-y

**Published:** 2018-08-01

**Authors:** Gavriil Shchedrin, Daniel Jaschke, Lincoln D. Carr

**Affiliations:** Colorado School of Mines, Golden, Colorado, 80401 USA

## Abstract

We explore the quantum many-body physics of a three-component Bose-Einstein condensate in an optical lattice driven by laser fields in *V* and Λ configurations. We obtain exact analytical expressions for the energy spectrum and amplitudes of elementary excitations, and discover symmetries among them. We demonstrate that the applied laser fields induce a gap in the otherwise gapless Bogoliubov spectrum. We find that Landau damping of the collective modes above the energy of the gap is carried by laser-induced roton modes and is considerably suppressed compared to the phonon-mediated damping endemic to undriven scalar condensates

## Introduction

**Figure 1:**
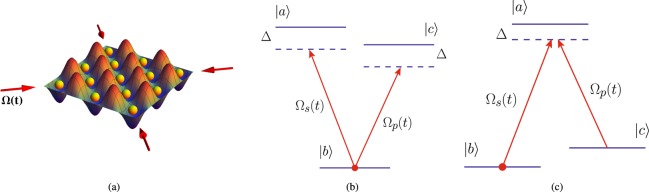
Three-Level Physics of BECs. (**a**) Multicomponent *F* = 1 BECs (depicted as yellow spheres) in optical lattices (depicted as a standing wave) can have internal states |*F*, *m*_*F*_〉 = {|*a*〉, |*b*〉, |*c*〉} driven by the laser fields Ω_*s*_(*t*) and Ω_*p*_(*t*) (depicted as red arrows) in the well-known (**b**) *V* and (**c**) Λ configurations. A laser field Ω_*s*_(*t*) drives the transition between the ground state $|b\rangle$ and the excited state $|a\rangle$, while Ω_*p*_(*t*) induces the transition between states $|c\rangle$ and $|b\rangle$ for a *V*-system and $|c\rangle$ and $|a\rangle$ for the Λ system. The laser fields are characterized by the Rabi frequencies Ω_*s*_ and Ω_*p*_ and equal detuning Δ from the excited states. Initially, the BEC is prepared in the ground state $|b\rangle$.

Multicomponent Bose-Einstein condensates (BECs) are a unique form of matter that allow one to explore coherent many-body phenomena in a macroscopic quantum system by manipulating its internal degrees of freedom^1–3^. The ground state of alkali-based BECs, which includes ^7^Li, ^23^Na, and ^87^Rb, is characterized by the hyperfine spin *F*, that can be best probed in optical lattices, which liberate its 2*F* + 1 internal components and thus provides a direct access to its internal structure^4–11^. Driven three-component *F* = 1 BECs in *V* and Λ configurations (see Fig. 1(b) and (c)) are totally distinct from two-component BECs^3^ due to the light interaction with three-level systems that results in the laser-induced coherence between excited states and ultimately leads to a number of fascinating physical phenomena, such as lasing without inversion (LWI)^12,13^, ultraslow light^14,15^, and quantum memory^16^. The key technique behind these phenomena is electromagnetically induced transparency (EIT)^13,17^, which is based on the elimination of real and imaginary parts of the susceptibility upon applying a coherent resonant drive to a gas of three-level atoms, that opens a transparency window in otherwise optically opaque atomic media^18–20^. The vanishing imaginary part of the susceptibility results in an extremely small group velocity of light, which led to the observation of unprecedented seven orders of magnitude slowdown of light propagation through a BEC of ^23^Na atoms^14^. The notion of non-dissipative dark-state polaritons not only yields a simple and elegant description of slow light phenomena^21–24^, but also provides an efficient way to store and retrieve individual quantum states, i.e., quantum memory^16,25–27^. Apart from physical phenomena achieved by the light-induced coherence in three-level systems, confined multicomponent BECs allowed the experimental realization of a number of fundamental physical concepts including the observation of the spin Hall effect^28^, creation of exotic magnetic^29^ and topological states^30,31^, and observation of Dirac monopoles^32^.

**Figure 2:**
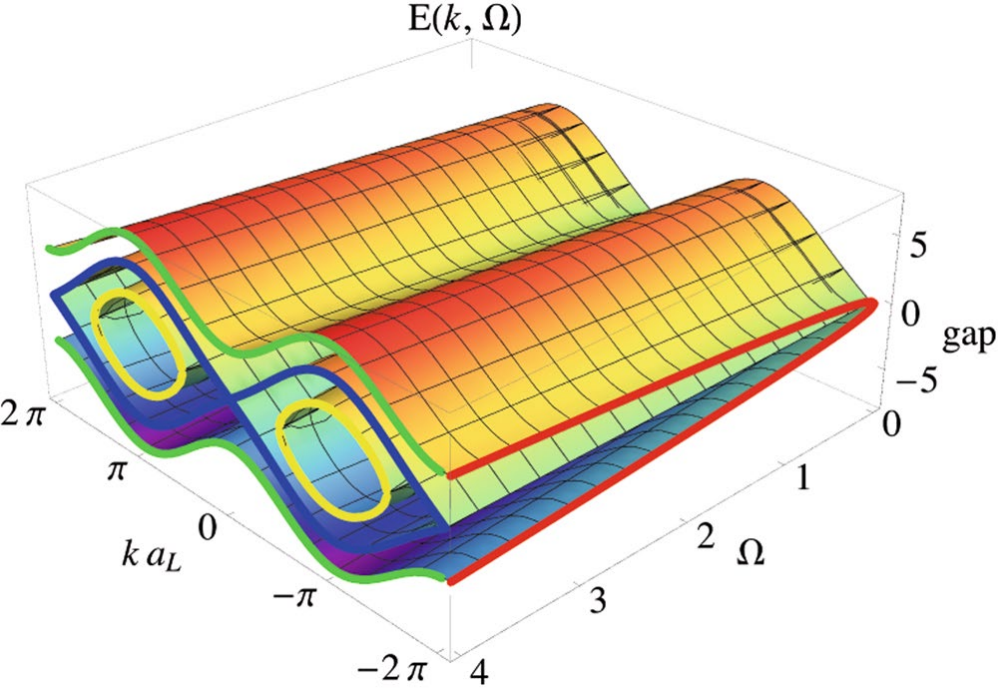
Rabi-Bogoliubov Spectrum. Energy gap for a 3-level BEC in *V* and Λ configurations as a function of driving frequency ${\rm{\Omega }}=\sqrt{{{\rm{\Delta }}}^{2}+{{\rm{\Omega }}}_{s}^{2}+{{\rm{\Omega }}}_{p}^{2}}$. Ω_*s*_ and Ω_*p*_ are the Rabi frequencies of the applied laser fields, and Δ is the detuning (see Fig. 1). The applied laser field induces a gap in the generalized Rabi-Bogoliubov spectrum (red curve). The Rabi-Bogoliubov spectrum is composed of three branches, separated by the standard gapless Bogoliubov spectrum (blue curve) linear at small momenta *k* (units of the lattice constant *a*_*L*_). The first branch of the generalized Rabi-Bogoliubov spectrum, which lies above the gap-less Bogoliubov spectrum (green curve) is characterized by the energy gap. The second branch of the Rabi-Bogoliubov spectrum (yellow curve) is characterized by the critical value of the momenta, below which it does not have real-valued solution, and therefore, is bounded by the standard Bogoliubov spectrum. We shall note that the Rabi-Bogoliubov energy spectrum is composed of the so-called positive and negative families of eigenmodes that are dual to each other. We adopt the common convention and consider the positive family of eigenstates that corresponds to the positive branch of the Rabi-Bogoliubov spectrum, i.e., $E(k,{\rm{\Omega }})\ge 0$ for all allowed momenta of the collective excitations.

However, access to these rich physical phenomena is limited or entirely excluded by the damping processes of the collective modes of the BEC^33–37^. The collective modes, or equivalently collective excitations, represent normal modes of the entire Bose-Einstein condensate. They mediate interaction in a many-body system of a BEC and are characterized with an energy and momentum and thus constitute the dispersion law of the entire BEC^3,38,39^. The damping of the collective modes manifests itself in the metastable nature of spinor BEC, which dictates its properties and the many-body phenomena governed by it. Suppression of damping processes for collective excitations has been previously discovered experimentally and described theoretically in several BEC contexts, e.g., absence of Beliaev damping, which governs the decay process of a single collective mode into two collective excitations of a lower energy, for a quasi-2D dipolar gas^33,34,40^; the Quantum Zeno mechanism, responsible for a diminished decay rate of collective excitations in a quantum degenerate fermionic gas of polar molecules confined in optical lattices^41^; and suppression of the Landau decay rate of collective excitations for a Bose-Fermi superfluid mixture^42,43^. However, in all these systems the energy spectrum is gapless for small momenta, and therefore, Landau damping is carried out predominantly by the phonons. In contrast to all these past studies, in this paper we calculate exact analytical expressions for the Landau damping rate in spinor three-component BECs in optical lattices driven by microwave fields in both *V* and Λ configurations. The resulting generalized Rabi-Bogoliubov (RB) energy spectrum, Rabi-Bogoliubov amplitudes, and symmetries among them allow us to explore near-equilibrium BEC dynamics. We find that the laser fields induce a gap (see Fig. 2) in the energy spectrum, preventing collective excitation from Landau damping, thus enabling a metastable state in a driven spinor BEC. The laser-induced gap in the energy spectrum results in zero group velocity and non-zero current for the collective modes lying above the energy of the gap. These collective modes mediate interaction of many-body interacting system of BEC and constitute the local minima of the dispersion law of the Rabi-Bogoliubov spectrum. Thus, these modes that we will refer as rotons, are characterized by the non-linear dispersion relation, as opposed to phonons that correspond to the linear dispersion relation of low-energy excitations in laser-free condensate^3,38,39^. We shall point out that in the long wave-length limit the spectrum of roton excitations turns into a free-particle spectrum plus the laser-induced gap. The laser-induced roton modes significantly suppress the Landau damping rate in spinor BECs compared to the phonon-mediated Landau damping in undriven scalar BECs.

## Results

### The Bose-Hubbard Hamiltonian

We begin with the second-quantized Hamiltonian for a driven three-component BEC,

1 $$\begin{array}{rcl}H & = & \int d\,{\bf{r}}\sum _{j=a,b,c}{\hat{\psi }}_{j}^{\dagger }({\bf{r}})(-\frac{{\hslash }^{2}}{2m}{\nabla }^{2}+V({\bf{r}})-{\mu }_{j}){\hat{\psi }}_{j}({\bf{r}})\\  &  & +\,\frac{1}{2}\int d\,{\bf{r}}\sum _{j=a,b,c}{\hat{\psi }}_{j}^{\dagger }({\bf{r}})(\sum _{j^{\prime} =a,b,c}{g}_{jj^{\prime} }{\hat{\psi }}_{j^{\prime} }^{\dagger }({\bf{r}}){\hat{\psi }}_{j^{\prime} }({\bf{r}})){\hat{\psi }}_{j}({\bf{r}})\\  &  & +\,\frac{{{\rm{\Omega }}}_{s}}{2}\int d\,{\bf{r}}\,({e}^{i{\rm{\Delta }}t}{\hat{\psi }}_{a}^{\dagger }({\bf{r}}){\hat{\psi }}_{b}({\bf{r}})+{e}^{-i{\rm{\Delta }}t}{\hat{\psi }}_{b}^{\dagger }({\bf{r}}){\hat{\psi }}_{a}({\bf{r}}))\\  &  & +\,\frac{{{\rm{\Omega }}}_{p}}{2}\int d\,{\bf{r}}\,({e}^{i{\rm{\Delta }}t}{\hat{\psi }}_{c}^{\dagger }({\bf{r}}){\hat{\psi }}_{b}({\bf{r}})+{e}^{-i{\rm{\Delta }}t}{\hat{\psi }}_{b}^{\dagger }({\bf{r}}){\hat{\psi }}_{c}({\bf{r}})),\end{array}$$

where we have chosen a *V*-configuration (see Fig. 1b) for concreteness. Here we introduced a Bose field operator ${\hat{\psi }}_{j}({\bf{r}})$, which annihilates a particle determined by the mass *m*, position ***r***, and internal state *j* = *b* (*a*, *c*) for a particle in the ground (excited) state. The lattice potential is assumed to have a simple cubic form, $V({\bf{r}})={V}_{0}\,{\sum }_{i=1}^{3}\,{\sin }^{2}({k}_{L}{r}_{i})$, where *k*_*L*_ = *π*/*a*_*L*_ is the lattice vector and *a*_*L*_ is the lattice constant. The coupling constant ${g}_{jj^{\prime} }$ determines the interaction between particles occupying the internal states *j* and $j^{\prime}$. The laser fields are characterized by the Rabi frequencies Ω_*s*_ and Ω_*p*_ and equal detuning Δ from excited states. Initially, the BEC is prepared in the ground state $|b\rangle$.

For sufficiently deep lattices, or alternatively in the long-wavelength approximation, one can safely adopt the lowest band approximation, and perform expansion of the bosonic field operators ${\hat{\psi }}_{j}({\bf{r}})$ in the Wannier basis ${\hat{\psi }}_{j}({\bf{r}})={\sum }_{n}\,{\hat{b}}_{nj}{w}_{j}({\bf{r}}-{{\bf{r}}}_{n})$. Throughout the paper, we will adopt the index convention, according to which the first argument of the field operator index denotes the site in an optical lattice, and the second argument indicates the internal state. Inserting this expansion into Eq. (1) we obtain,

2 $$\begin{array}{rcl}H & = & -\sum _{j=a,b,c}\sum _{\langle m,n\rangle }\,{J}_{mn}^{jj}({\hat{b}}_{mj}^{\dagger }{\hat{b}}_{nj}+{\hat{b}}_{nj}^{\dagger }{\hat{b}}_{mj})-\sum _{j=a,b,c}\,{\mu }_{j}\,\sum _{n}\,{\hat{b}}_{nj}^{\dagger }{\hat{b}}_{nj}+\sum _{j,j^{\prime} =a,b,c}\,\frac{{U}_{jj^{\prime} }}{2}\,\sum _{n}\,{\hat{b}}_{nj}^{\dagger }{\hat{b}}_{nj^{\prime} }^{\dagger }{\hat{b}}_{nj^{\prime} }{\hat{b}}_{nj}\\  &  & +\,\frac{{{\rm{\Omega }}}_{s}}{2}\,\sum _{n}\,({e}^{i{\rm{\Delta }}t}{\hat{b}}_{na}^{\dagger }{\hat{b}}_{nb}+{e}^{-i{\rm{\Delta }}t}{\hat{b}}_{nb}^{\dagger }{\hat{b}}_{na})+\frac{{{\rm{\Omega }}}_{p}}{2}\,\sum _{n}\,({e}^{i{\rm{\Delta }}t}{\hat{b}}_{nc}^{\dagger }{\hat{b}}_{nb}+{e}^{-i{\rm{\Delta }}t}{\hat{b}}_{nb}^{\dagger }{\hat{b}}_{nc}),\end{array}$$

where we truncated the sum to the nearest neighbors, indicated by $\langle m,n\rangle$. Here the hopping integral is

3 $${J}_{mn}^{ij}=-\,\int \,d\,{\bf{r}}{w}_{i}^{\ast }({\bf{r}}-{{\bf{r}}}_{m})\,[-\frac{{\hslash }^{2}}{2m}{\nabla }^{2}+V({\bf{r}})]\,{w}_{j}^{\ast }({\bf{r}}-{{\bf{r}}}_{n}),$$

the on-site interaction is

4 $${U}_{jj^{\prime} }={g}_{jj^{\prime} }\int d{\bf{r}}{w}_{j}^{\ast }({\bf{r}}){w}_{j^{\prime} }^{\ast }({\bf{r}}){w}_{j^{\prime} }({\bf{r}}){w}_{j}({\bf{r}}).$$

In order to formulate Eq. (2) in *k*-space we introduce the Fourier transform of the creation and annihilation operators,

5 $${\hat{b}}_{nj}=\frac{1}{\sqrt{{N}_{L}}}\,\sum _{k}\,\exp [\,-\,i{\bf{k}}{{\bf{r}}}_{n}]{\hat{a}}_{kj},$$

where *N*_*L*_ is number of lattice sites. In order to linearize the Fourier-transformed Hamiltonian given by Eq. (2) we expand the operators near their average values, i.e., ${\hat{a}}_{kj}=\langle {\hat{a}}_{0j}\rangle +({\hat{a}}_{kj}-\langle {\hat{a}}_{0j}\rangle )$. The average value of the field operator $\langle {\hat{a}}_{0j}\rangle$ is given in terms of the number of particles occupying the zero momentum state *N*_0*j*_, i.e., $\langle {\hat{a}}_{0j}\rangle =\sqrt{{N}_{0j}}$. The linearized Hamiltonian becomes,

6 $$\begin{array}{ccc}{H}_{{\rm{e}}{\rm{f}}{\rm{f}}} & = & \frac{1}{2}\,\sum _{k}\,({a}_{k,j=a}^{\dagger },{a}_{k,j=b}^{\dagger },{a}_{k,j=c}^{\dagger },{a}_{-k,j=a},{a}_{-k,j=b},{a}_{-k,j=c})\\  &  & \times (\begin{array}{cccccc}{h}_{k}+{\rm{\Delta }} & -\frac{{{\rm{\Omega }}}_{s}}{2} & 0 & u & s & t\\ -\frac{{{\rm{\Omega }}}_{s}}{2} & {h}_{k} & -\frac{{{\rm{\Omega }}}_{p}}{2} & s & u & s\\ 0 & -\frac{{{\rm{\Omega }}}_{p}}{2} & {h}_{k}+{\rm{\Delta }} & t & s & u\\ u & s & t & {h}_{k}+{\rm{\Delta }} & -\frac{{{\rm{\Omega }}}_{s}}{2} & 0\\ s & u & s & -\frac{{{\rm{\Omega }}}_{s}}{2} & {h}_{k} & -\frac{{{\rm{\Omega }}}_{p}}{2}\\ t & s & u & 0 & -\frac{{{\rm{\Omega }}}_{p}}{2} & {h}_{k}+{\rm{\Delta }}\end{array})(\begin{array}{c}{a}_{k,j=a}\\ {a}_{k,j=b}\\ {a}_{k,j=c}\\ {a}_{-k,j=a}^{\dagger }\\ {a}_{-k,j=b}^{\dagger }\\ {a}_{-k,j=c}^{\dagger }\end{array}).\end{array}$$

Here, the tunneling parameter *h*_*k*_ = *u* + *t*_*k*_, with ${t}_{k}=4J{\sin }^{2}(k{a}_{L}\mathrm{/2})$, is given in terms of the tunneling amplitude $J\equiv {J}_{mn}^{jj}$, momentum *k*, and the lattice constant *a*_*L*_. The matrix which describes the interaction between the BEC’s constituents that are characterized by the momentum *k* and the internal state *j* = {*a*, *b*, *c*}, is given by

7 $$(\begin{array}{ccc}u & s & t\\ s & u & s\\ t & s & u\end{array})\equiv (\begin{array}{ccc}{n}_{a}{U}_{aa} & \sqrt{{n}_{a}{n}_{b}}{U}_{ab} & \sqrt{{n}_{a}{n}_{c}}{U}_{ac}\\ \sqrt{{n}_{b}{n}_{a}}{U}_{ba} & {n}_{b}{U}_{bb} & \sqrt{{n}_{b}{n}_{c}}{U}_{ac}\\ \sqrt{{n}_{c}{n}_{a}}{U}_{ca} & \sqrt{{n}_{c}{n}_{b}}{U}_{cb} & {n}_{c}{U}_{cc}\end{array}).$$

The coupling matrix in Eq. (6) is given up to the leading order of the average filling factor *n*_*j*_ = *N*_0*j*_/*N*_*L*_ for the particles characterized by the internal state *j* and momentum *k* = 0. In realistic three-component BECs one cannot neglect excitations that correspond to the interactions occupying excited states *j* = *a* and *j* = *c*. The main goal of the present paper, however, is to present an *analytical model* of a laser-driven three-component BEC that allows one to obtain *analytical* expressions for the Rabi-Bogoliubov energy spectrum and Rabi-Bogoliubov amplitudes. This allows one to evaluate the Landau damping of collective modes that unequivocally shows that collective modes below the energy of the laser-induced gap remain undamped, while above the gap is governed by the rotons and is significantly suppressed as compared to the phonon-mediated laser-free scalar BEC. Thus, in this paper we have adopted the simplest non-trivial case that correspond to *s* = 0 and *t* = 0. We have analyzed the most general case *s* ≠ 0 and *t* ≠ 0 numerically and found that the main physical results concerning the gapped structure of the energy spectrum, the amplitudes of elementary excitations of a driven three-component BEC, and symmetries among them, obtained in the most general case match the present description. However, in this case we cannot obtain analytical results for the Rabi-Bogoliubov energy spectrum and amplitudes, which thus prevents us from analytical calculation of the Landau damping. Since the primary goal of the present paper is to present analytical model of a driven three-component BEC in an optical lattice, the general case goes beyond the scope of this approach.

**Figure 3:**
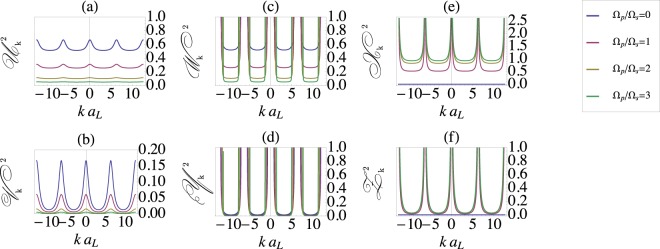
Rabi-Bogoliubov Amplitudes as a Function of Rabi Frequency. The standard Bogoliubov amplitudes have poles at the location of the roots of Bogoliubov spectrum. The laser field, which drives the BEC, creates a gap in the spectrum of elementary excitations. As a result, Rabi-Bogoliubov amplitudes (**a**) ${{\mathscr{U}}}_{k}^{2}$ and (**b**) ${{\mathscr{V}}}_{k}^{2}$ are finite for all values of momenta *k*, given in the units of the lattice constant *a*_*L*_.

The diagonalization of the Fourier-transformed Bose-Hubbard Hamiltonian can be accomplished via the generalized Bogoliubov transformation. This transformation is carried out by the quasi-particle operators ${\hat{\alpha }}_{k,a}$, ${\hat{\zeta }}_{kc}$, and ${\hat{\beta }}_{k,b}$, that annihilate a particle occupying the internal state *j* = *a*, *j* = *c* (excited states) and *j* = *b* (ground state), correspondingly. The transformation from the particle to the quasi-particle basis is given by the linear combination, ${\hat{a}}_{kj}={{\mathscr{U}}}_{k}{\hat{\alpha }}_{ka}+{{\mathscr{V}}}_{k}^{\ast }{\hat{\alpha }}_{-ka}^{\dagger }+{{\mathscr{W}}}_{k}{\hat{\beta }}_{kb}+{{\mathscr{Y}}}_{k}^{\ast }{\hat{\beta }}_{-kb}^{\dagger }+{{\mathscr{X}}}_{k}{\hat{\zeta }}_{kc}+{{\mathscr{Z}}}_{k}^{\ast }{\hat{\zeta }}_{-kc}^{\dagger }$. We shall provide a clear physical picture of the transformation from the particle to quasi-particle basis in a driven three-component BEC. We recall that in a scalar BEC, creation and annihilation operators of the excitations that carry momentum ***k*** are given in terms of a linear superposition of quasi-particle creation and annihilation operators that carry both positive and negative momenta. The three-component BECs in optical lattices have three extra degrees of freedom that correspond to the internal states of a driven BEC, $|F,{m}_{F}\rangle =\{|a\rangle ,\,|b\rangle ,\,|c\rangle \}$ (see. Fig. 1). Thus, creation and annihilation operators in a driven three-component BEC are given by a linear superposition of six quasiparticle operators. These operators carry the excitations in either of the three internal states of a driven three-component BEC with both positive and negative momenta. The generalized Rabi-Bogoliubov amplitudes (see Fig. 3) are subjected to a constraint ${{\mathscr{U}}}_{k}^{2}-{{\mathscr{V}}}_{k}^{2}+{{\mathscr{W}}}_{k}^{2}-{{\mathscr{Y}}}_{k}^{2}+{{\mathscr{X}}}_{k}^{2}-{{\mathscr{Z}}}_{k}^{2}=1$, which ensures the bosonic commutation relation for the quasi-particle creation and annihilation operators, i.e., $[{\hat{\alpha }}_{kj},{\hat{\alpha }}_{-k,j^{\prime} }^{\dagger }]={\delta }_{jj^{\prime} }$, and $[{\hat{\beta }}_{kj},{\hat{\beta }}_{-k,j^{\prime} }^{\dagger }]={\delta }_{jj^{\prime} }$, and $[{\hat{\zeta }}_{kj},{\hat{\zeta }}_{-k,j^{\prime} }^{\dagger }]={\delta }_{jj^{\prime} }$.

### The generalized Rabi-Bogoliubov spectrum and amplitudes

Now we turn to the calculation of the energy spectrum in driven three-component condensates in both *V* and Λ configurations. In the basis of quasiparticle creation and annihilation operators, the Bose-Hubbard Hamiltonian acquires a diagonal form,

8 $${H}_{{\rm{eff}}}=\frac{1}{2}\sum _{k}({E}_{a}(k){\hat{\alpha }}_{a,k}^{\dagger }{\hat{\alpha }}_{a,k}+{E}_{b}(k){\hat{\beta }}_{b,k}^{\dagger }{\hat{\beta }}_{b,k}+{E}_{c}(k){\hat{\zeta }}_{b,k}^{\dagger }{\hat{\zeta }}_{b,k}),$$

where *E*_*a*_(*k*), *E*_*b*_(*k*), and *E*_*c*_(*k*) are the three branches of the Rabi-Bogoliubov energy spectrum, see Fig. 2, for both *V* and Λ configurations. In the case of the *V*-configuration, the energy spectrum is obtained from the condition *det*[*M*_*V*_−**1**(*E*/2)] = 0, where

9 $${M}_{V}=(\begin{array}{cccccc}{h}_{k}+{\rm{\Delta }} & -\frac{{{\rm{\Omega }}}_{s}}{2} & 0 & u & 0 & 0\\ -\frac{{{\rm{\Omega }}}_{s}}{2} & {h}_{k} & -\frac{{{\rm{\Omega }}}_{p}}{2} & 0 & u & 0\\ 0 & -\frac{{{\rm{\Omega }}}_{p}}{2} & {h}_{k}+{\rm{\Delta }} & 0 & 0 & u\\ -u & 0 & 0 & -{h}_{k}-{\rm{\Delta }} & \frac{{{\rm{\Omega }}}_{s}}{2} & 0\\ 0 & -u & 0 & \frac{{{\rm{\Omega }}}_{s}}{2} & -{h}_{k} & \frac{{{\rm{\Omega }}}_{p}}{2}\\ 0 & 0 & -u & 0 & \frac{{{\rm{\Omega }}}_{p}}{2} & -{h}_{k}-{\rm{\Delta }}\end{array}).$$

Here, the tunneling parameter *h*_*k*_ = *u* + *t*_*k*_, with ${t}_{k}=4J{\sin }^{2}(k{a}_{L}\mathrm{/2})$, is given in terms of the tunneling amplitude $J\equiv {J}_{mn}^{jj}$, momentum *k*, and the lattice constant *a*_*L*_. Thus, the Rabi-Bogoliubov spectrum is

10 $$\begin{array}{ccc}{E}_{a,\pm }^{V}(k) & = & \pm \sqrt{4{\varepsilon }_{k}^{2}+4({t}_{k}+u){\rm{\Delta }}+{{\rm{\Delta }}}^{2}+{{\rm{\Omega }}}^{2}+2\sigma },\\ {E}_{b,\pm }^{V}(k) & = & \pm \sqrt{4{\varepsilon }_{k}^{2}+4({t}_{k}+u){\rm{\Delta }}+{{\rm{\Delta }}}^{2}+{{\rm{\Omega }}}^{2}-2\sigma },\\ {E}_{c,\pm }^{V}(k) & = & \pm \sqrt{({t}_{k}+{\rm{\Delta }})({t}_{k}+2u+{\rm{\Delta }})},\end{array}$$

where ${\varepsilon }_{k}=\sqrt{{t}_{k}({t}_{k}+2u)}$ is the standard Bogoliubov spectrum^3^, ${\rm{\Omega }}=\sqrt{{{\rm{\Delta }}}^{2}+{{\rm{\Omega }}}_{s}^{2}+{{\rm{\Omega }}}_{p}^{2}}$ is the effective Rabi frequency of the combined laser fields Ω_*s*_(*t*) and Ω_*p*_(*t*), and $\sigma \equiv (2({t}_{k}+u)+{\rm{\Delta }}){\rm{\Omega }}$. We shall note that the Rabi-Bogoliubov energy spectrum, given by Eq. (10), is comprised of the so-called positive and negative families of eigenmodes^44^. The positive family of eigenstates corresponds to the positive branch of the Rabi-Bogoliubov spectrum, i.e., $E(k)\ge 0$ for all allowed momenta of the collective excitations *k*, which is dual to the negative family of the eigenstates. Moreover, the eigenspace of the nagative family is spanned by the eigenvectors that are complex conjugate of the eigenvectors that constitue the positive family^44^. Unless otherwise specified, we will concentrate exclusively on the positive family of eigenstates, i.e., we choose the positive branch of the Rabi-Bogoliubov spectrum for both *V* and Λ configurations of BECs.

We find a set of new symmetries that holds among the generalized Rabi-Bogoliubov amplitudes,

11 $$\begin{array}{rcl}{{\mathscr{V}}}_{k}^{2}({E}_{a},{\rm{\Omega }}) & = & -{{\mathscr{U}}}_{k}^{2}(\,-\,{E}_{a},{\rm{\Omega }}),\\ {{\mathscr{W}}}_{k}^{2}({E}_{b},{\rm{\Omega }}) & = & {{\mathscr{U}}}_{k}^{2}({E}_{b},-\,{\rm{\Omega }}),\\ {{\mathscr{Y}}}_{k}^{2}({E}_{b},{\rm{\Omega }}) & = & -{{\mathscr{U}}}_{k}^{2}(\,-\,{E}_{b},-{\rm{\Omega }}),\\ {{\mathscr{X}}}_{k}^{2}({E}_{c}) & = & -{{\mathscr{Z}}}_{k}^{2}(\,-\,{E}_{c}).\end{array}$$

The new symmetries summarized by Eq. (11) are the direct generalization of the intrinsic symmetries of the standard Bogoliubov amplitudes, i.e., ${u}_{k}^{2}(E)=-\,{v}_{k}^{2}(\,-\,E)$^3^. These symmetries generate the complete set of the generalized Rabi-Bogoliubov amplitudes from a single amplitude ${{\mathscr{U}}}_{k}^{2}({E}_{a},{\rm{\Omega }})$, which for a *V*-system is explicitly given by

12 $${{\mathscr{U}}}_{k}^{2}({E}_{a},{\rm{\Omega }})=\frac{{{\rm{\Omega }}}_{p}^{2}+{{\rm{\Omega }}}_{s}^{2}+(2({t}_{k}+u)+{E}_{a}^{{\rm{V}}})({\rm{\Omega }}-{\rm{\Delta }})}{4{E}_{a}^{{\rm{V}}}{\rm{\Omega }}},$$

and for the Λ-system,

13 $${{\mathscr{U}}}_{k}^{2}({E}_{a},{\rm{\Omega }})=\frac{{{\rm{\Omega }}}_{s}^{2}[{{\rm{\Omega }}}_{p}^{2}+{{\rm{\Omega }}}_{s}^{2}+(2({t}_{k}+u)+{E}_{a}^{{\rm{\Lambda }}})({\rm{\Omega }}-{\rm{\Delta }})]}{4{E}_{a}^{{\rm{\Lambda }}}({{\rm{\Omega }}}_{p}^{2}+{{\rm{\Omega }}}_{s}^{2}){\rm{\Omega }}}.$$

The symmetries in the *V*-system result in cancellation of the amplitudes ${{\mathscr{X}}}_{k}({E}_{c})$ and ${{\mathscr{Z}}}_{k}({E}_{c})$, while for the Λ-system we have

14 $${{\mathscr{X}}}_{k}^{2}({E}_{c})=\frac{{{\rm{\Omega }}}_{p}^{2}({t}_{k}+u+{E}_{c}^{{\rm{\Lambda }}})}{2{E}_{c}^{{\rm{\Lambda }}}({{\rm{\Omega }}}_{p}^{2}+{{\rm{\Omega }}}_{s}^{2})}.$$

Here ${E}_{a}^{{\rm{\Lambda }}}(k)$, ${E}_{b}^{{\rm{\Lambda }}}(k)$, and ${E}_{c}^{{\rm{\Lambda }}}(k)$ are solutions of the eigenvalue problem for the Λ configuration governed by

15 $${M}_{{\rm{\Lambda }}}=(\begin{array}{cccccc}{h}_{k}+{\rm{\Delta }} & -\frac{{{\rm{\Omega }}}_{s}}{2} & -\frac{{{\rm{\Omega }}}_{p}}{2} & u & 0 & 0\\ -\frac{{{\rm{\Omega }}}_{s}}{2} & {h}_{k} & 0 & 0 & u & 0\\ -\frac{{{\rm{\Omega }}}_{p}}{2} & 0 & {h}_{k} & 0 & 0 & u\\ -u & 0 & 0 & -{h}_{k}-{\rm{\Delta }} & \frac{{{\rm{\Omega }}}_{s}}{2} & \frac{{{\rm{\Omega }}}_{p}}{2}\\ 0 & -u & 0 & \frac{{{\rm{\Omega }}}_{s}}{2} & -{h}_{k} & 0\\ 0 & 0 & -u & \frac{{{\rm{\Omega }}}_{p}}{2} & 0 & -{h}_{k}\end{array}).$$

The eigenvalues in Λ-configuration are given in terms of the energy spectrum for the *V*-system, Eq. (10),

16 $$\begin{array}{rcl}{E}_{a}^{{\rm{\Lambda }}}(k) & = & {E}_{a}^{V}(k),\\ {E}_{b}^{{\rm{\Lambda }}}(k) & = & {E}_{b}^{V}(k),\\ {E}_{c,\pm }^{{\rm{\Lambda }}}(k) & = & \pm \sqrt{{t}_{k}({t}_{k}+2u)}.\end{array}$$

In the long wavelength limit the Rabi-Bogoliubov amplitudes ${{\mathscr{W}}}_{k}$, ${{\mathscr{Y}}}_{k}$ are purely imaginary. Therefore, we are left with the real-valued Rabi-Bogoliubov amplitudes ${{\mathscr{U}}}_{k}(E)$, ${{\mathscr{V}}}_{k}(E)$, ${{\mathscr{X}}}_{k}(E)$, and ${{\mathscr{Z}}}_{k}(E)$, that could be further simplified in case of resonant driving fields, i.e., Δ = 0. For the laser fields in the *V*-configuration we have,

17 $$\begin{array}{rcl}{{\mathscr{U}}}_{k}^{V}({E}_{a}) & = & \sqrt{[{E}_{a}+\mathrm{2(}{t}_{k}+u+{{\rm{\Omega }}}_{0}\mathrm{/2)]/(4}{E}_{a})},\\ {{\mathscr{V}}}_{k}^{V}({E}_{a}) & = & -\sqrt{[\,-\,{E}_{a}+\mathrm{2(}{t}_{k}+u+{{\rm{\Omega }}}_{0}\mathrm{/2)]/(4}{E}_{a})},\end{array}$$

while for the Λ-system the Rabi-Bogoliubov amplitudes can be simplified into,

18 $$\begin{array}{rcl}{{\mathscr{U}}}_{k}^{{\rm{\Lambda }}}({E}_{a}) & = & \frac{{{\rm{\Omega }}}_{s}}{{{\rm{\Omega }}}_{0}}\sqrt{\frac{{E}_{a}+\mathrm{2(}{t}_{k}+u+{{\rm{\Omega }}}_{0}\mathrm{/2)}}{4{E}_{a}}},\\ {{\mathscr{V}}}_{k}^{{\rm{\Lambda }}}({E}_{a}) & = & -\frac{{{\rm{\Omega }}}_{s}}{{{\rm{\Omega }}}_{0}}\sqrt{\frac{-{E}_{a}+\mathrm{2(}{t}_{k}+u+{{\rm{\Omega }}}_{0}\mathrm{/2)}}{4{E}_{a}}},\end{array}$$

where we have chosen the real-valued Rabi-Bogoliubov amplitudes for both *V* and Λ configurations of a driven BEC. The effective Rabi frequency simplifies into $${{\rm{\Omega }}}_{0}\equiv {\rm{\Omega }}({\rm{\Delta }}=0)=\sqrt{{{\rm{\Omega }}}_{s}^{2}+{{\rm{\Omega }}}_{p}^{2}}$$ and $${E}_{a}=\sqrt{(2{t}_{k}+{\rm{\Omega }})(2{t}_{k}+4u+{\rm{\Omega }})}$$ and $${E}_{c}^{{\rm{\Lambda }}}=\sqrt{{t}_{k}({t}_{k}+2u)}$$. The amplitudes $${{\mathscr{X}}}_{k}^{{\rm{\Lambda }}}({E}_{c})={{\rm{\Omega }}}_{p}/{{\rm{\Omega }}}_{0}{u}_{k}({E}_{c})$$ and $${{\mathscr{X}}}_{k}^{{\rm{\Lambda }}}({E}_{c})={{\rm{\Omega }}}_{p}/{{\rm{\Omega }}}_{0}{v}_{k}({E}_{c})$$ are given in terms of the standard Bogoliubov amplitudes *u*_*k*_ and *v*_*k*_. The standard Bogoliubov amplitudes *u*_*k*_ and *v*_*k*_ are defined in terms of the Bogoliubov spectrum, $${\varepsilon }_{k}=\sqrt{{t}_{k}({t}_{k}+2u)}$$, and are given by $${u}_{k}=\sqrt{({t}_{k}+u+{\varepsilon }_{k})/(2{\varepsilon }_{k})}$$ and $${v}_{k}=$$$$-\sqrt{({t}_{k}+u-{\varepsilon }_{k})/(2{\varepsilon }_{k})}$$^3,45,46^. Mathematically, Bogoliubov amplitudes constitute a rotation matrix that brings the Bose-Hubbard Hamiltonian of a scalar BEC into a diagonal form. Physically, Bogoliubov amplitudes transform a system of interacting particles that constitutes a scalar BEC into a system of non-interacting quasi-particles^3,38^.

The obtained Rabi-Bogoliubov energy spectrum and amplitudes of elementary excitations describe driven three-component BECs in an optical lattice, such as ^7^Li-, ^23^Na-, and ^87^Rb-based BECs^4–11^. As we pointed out earlier, the optical lattice liberates internal components of driven BECs, and thus allows a direct access to their internal structure. However, the obtained results can be immediately applied in the special case of a driven three-component homogeneous BEC. Indeed, in the long-wavelength limit, the kinetic energy given by ${t}_{k}=4J\,{\sin }^{2}(k{a}_{L}\mathrm{/2})$ can be simplified into ${t}_{k}\simeq J{a}_{L}^{2}{k}^{2}\equiv {k}^{2}/(2{m}^{\ast })$, which is nothing but the free-particle dispersion relation of the homogeneous BEC^3^.

We shall point out that the present method of getting the exact Rabi-Bogoliubov energy spectrum and amplitudes of elementary excitations is based on the minimization of the expectation value of the Bose-Hubbard Hamiltonian with respect to a large number of atoms in the condensate. At the minimum, the terms linear with respect to particle creation and annihilation operators in the Bose-Hubbard Hamiltonian must vanish, which ensures the absence of fluctuations^3,47^. Thus, the minimization procedure leads to a quadratic form of the many-body Bose-Hubbard Hamiltonian, which can be diagonalized in the quasi-particle basis by means of the generalized Bogoliubov transformation^3,47^. We shall stress, however, that this method is completely equivalent to the approach based on the linearization of the Gross-Pitaevskii equation formulated in terms of the condensate wave function^3,44^. The latter method results in a system of Bogoliubov equations in the leading order of the perturbation to the condensate wave function and its complex conjugate. The system of coupled Bogoliubov equations immediately leads to the energy spectrum and amplitudes of elementary excitations that agree with the amplitudes obtained from the linearization of the Bose-Hubbard Hamiltonian given by Eq. (1). The reason we have chosen the Hamiltonian approach, as opposed to the Gross-Pitaevskii equation, is that it allows the direct application of the microscopic formalism of Landau damping in BECs^48^. The operator method provides the consistent derivation of the matrix element of the process of scattering of a collective mode by a thermal mode, which would be impossible to obtain from the Gross-Pitaevskii equation. The following section is dedicated to the calculation of the Landau damping of a collective mode in a driven three-component BEC.

### Landau damping of collective modes in three-component condensates

In this section we will concentrate on the interaction between the collective modes and thermally excited modes in a driven three-component BEC. Specifically, we will consider the scattering of an incoming collective mode and a thermal mode that results in a single outgoing thermal mode, known as the 2−1 process^3,48^. This process results in Landau damping^49^ of collective excitations in BECs. We shall point out that in the 2−1 process, thermal excitations may gain or lose its energy, which ultimately depends on their thermal distribution. As we will see, the Landau damping rate depends on the slope of the thermal distribution, which is negative for the Bose-Einstein distribution. Thus, thermal modes experience a net loss of energy which ultimately results in Landau damping of collective excitations^3^.

Now we turn to the calculation of the Landau damping of collective modes in driven three-component condensates in both *V* and Λ configurations. Introducing *E* = *E*_*a*_/2, we obtain the following expression for the Landau damping rate for the laser fields in the *V* configuration,

19 $${{\rm{\Gamma }}}_{L}^{V}=-\pi \hslash {\omega }_{q}\frac{2\pi }{{\mathrm{(2}\pi \hslash )}^{3}}{(4\sqrt{N}\frac{{g}_{jj}}{2}\frac{\sqrt{{\omega }_{q}}}{\sqrt{2}\sqrt{u+s}})}^{2}\frac{1}{q}\beta \frac{\partial }{\partial \beta }\int dp\,\frac{1}{{v}_{g}}\frac{{p}^{2}}{E}\frac{1}{({e}^{\beta E}-\mathrm{1)}}{(\frac{3}{4}\frac{E}{(u+s)})}^{2}.$$

Here *ω*_*q*_ and *q* are the frequency and momentum of the collective mode, respectively. In the Λ-configuration the Landau damping rate is

20 $${{\rm{\Gamma }}}_{L}^{{\rm{\Lambda }}}={({{\rm{\Omega }}}_{s}/{{\rm{\Omega }}}_{0})}^{2}{{\rm{\Gamma }}}_{L}^{{\rm{V}}}+{({{\rm{\Omega }}}_{p}/{{\rm{\Omega }}}_{0})}^{2}{{\rm{\Gamma }}}_{L},$$

where Γ_*L*_ is the usual Landau damping rate in the laser-free case^48^. The Landau damping acquires a particularly simple form if we introduce the density of the laser-induced roton modes,

21 $${\rho }_{r}=\frac{4\pi }{\mathrm{3(2}\pi \hslash {)}^{3}}(-\beta \frac{\partial }{\partial \beta }){\int }_{{E}_{0}}^{\infty }dE\,\frac{{p}^{2}}{{v}_{g}^{2}}\frac{E}{({e}^{\beta E}-1)}.$$

Here we have introduced the group velocity *v*_*g*_ = ∂*E*(*p*)/∂*p*, the Boltzmann factor *β* = 1/*k*_*B*_*T*, and the Boltzmann constant *k*_*B*_. The Taylor expansion of the energy around zero momentum returns, $E\simeq {E}_{0}+{E}_{2}\,{p}^{2}/2$, where the gap in the spectrum is ${E}_{0}=\sqrt{{\rm{\Omega }}(4u+{\rm{\Omega }})}/2$, and the curvature of the spectrum is ${E}_{2}=(2u+{\rm{\Omega }})/[{m}^{\ast }\sqrt{{\rm{\Omega }}(4u+{\rm{\Omega }})}]$. The effective mass is given by ${m}^{\ast }=1/(J{a}_{L}^{2})$.

Finally, we can express the rate of Landau damping for a three-component BEC driven by the laser fields in a *V*-configuration in terms of the density of the laser-induced roton modes,

22 $${{\rm{\Gamma }}}_{L}^{V}=\theta (\hslash {\omega }_{q}-{E}_{0})\frac{27\pi }{16}\hslash {\omega }_{q}\frac{{\rho }_{r}}{\rho ({\omega }_{q})}.$$

Here the spectral density of the collective modes, $\rho ({\omega }_{q})=q{(u+s)}^{3}/({g}_{jj}^{2}N{\omega }_{q})$, is given in terms the $q=\sqrt{2(\hslash {\omega }_{q}-{E}_{0})/{E}_{2}}$. We immediately find that the collective modes characterized by the energy not exceeding the energy of the gap ($\hslash {\omega }_{q} < {E}_{0}$) are free from Landau damping, i.e., Γ_*L*_ = 0. Therefore, the gap in the energy spectrum produced by the applied laser fields effectively protects low-lying collective modes from Landau damping. For the collective modes lying above the energy of the gap, the Landau damping rate scales with the density of the laser-induced roton modes, which in the limit of low temperatures behaves as ${\rho }_{r}\simeq {\beta }^{-2}$. In the limiting case of laser-free condensate, i.e., Ω_*s*_ = Ω_*p*_ = 0, the Rabi-Bogoliubov spectrum simplifies to the standard Bogoliubov spectrum. As a result, the laser-modified Landau damping rate Eq. (21) reduces to the well-known result^48^ for the phonon-mediated Landau damping of the collective modes in scalar BEC, ${{\rm{\Gamma }}}_{L}({{\rm{\Omega }}}_{s}={{\rm{\Omega }}}_{p}=0)=\frac{27\pi }{16}\hslash {\omega }_{q}\frac{{\rho }_{n}}{\rho }\simeq \frac{1}{{\beta }^{4}}$, defined in terms of the density of a phonon gas *ρ*_*n*_ = 2*π*^2^*T*^4^/(45ℏ^3^*c*^5^)^38^. Thus, the Landau damping rate of the collective excitations in a driven three-level BEC is significantly slowed down compared to scalar laser-free BEC, where damping processes are mediated by phonons.

### Discussions and Conclusions

Experimentally, the absence of Landau damping in driven three-component condensate can be verified by means of the two-photon Bragg spectroscopy. This technique was successfully applied in measuring Beliaev damping of the collective modes^50^, which revealed a complete absence of the collision of quasiparticles below a critical momentum in a BEC of ^87^Rb atoms. In case of Beliaev damping of collective modes in a laser-free BEC, as well as in case of Landau damping in a laser-driven spinor condensate, both physical systems are characterized by a critical energy, below which collision of the collective modes and the corresponding damping processes are entirely excluded. Thus, we conclude that despite the fact that the collision of the quasiparticles reported in the experiment^50^ was governed by Beliaev damping, we anticipate the same results for Landau damping of collective modes in a laser-driven three-component spinor Bose-Einstein condensates.

In conclusion, we investigated the quantum many-body physics of a three-component BEC confined in optical lattices and driven by laser fields in both *V* and Λ configurations. We found that the applied laser fields create a gap in the spectrum that shields collective excitation of the condensate lying below the energy of the gap from Landau damping. Above the gap, Landau damping is proportional to the density of the laser-induced roton modes, and is substantially suppressed compared to the Landau damping rate in an undriven scalar condensate carried by the phonons. This advance provides a prescription for the realization of electromagnetically induced transparency and other exciting three-level phenomena in multicomponent Bose-Einstein condensates.
